# Education and social care predictors of offending trajectories: A UK administrative data linkage study

**DOI:** 10.23889/ijpds.v8i2.2206

**Published:** 2023-09-14

**Authors:** Hannah Dickson, George Vamvakas, Roxanna Short, Nigel Blackwood

**Affiliations:** 1 King's College London, London, United Kingdom

## Objectives

The age-crime curve indicates that criminal behaviour peaks in adolescence and decreases in adulthood, but longitudinal studies suggest that this curve conceals distinct patterns of (re)-offending or trajectories. Some trajectories (e.g., life course persistent offenders) are reported to have distinct risk factors and more negative outcomes than others (e.g., adolescent limited offenders).

## Method

The current study had two main objectives: (1) To use UK administrative crime data to identify trajectories of (re)-offending; and (2) To prospectively identify (re)-offending trajectories using longitudinal administrative education and social care data. This project uses linked UK administrative data containing the anonymised education and social care records for individuals born between September 1985 and August 1999, which have been linked to later official crime records up to the end of 2017. To identify offending trajectories, we used information on offence type, age of first conviction/caution, age of last recorded conviction/caution and offending history at three age points (Juvenile: 10-17 years; Young adult: 18-20 years; Adult: 21-32 years).

## Results

Latent Class Analyses with and without ‘Gender’ and ‘Ever served a custodial sentence’ as covariates was conducted to identify trajectories of (re)-offending. We are currently developing statistical models to see if we can use prospective longitudinal education and social care factors to discriminate between these trajectories. In my talk, I will share findings on the offending trajectories identified and present some early results on the key education and social care drivers of the offending trajectories.

## Conclusion

Findings from this study has the potential to provide deeper insights into how these education and social care factors might affect (re)-offending patterns. This could inform education, social care and criminal justice system responses to offending behaviours which seek to reduce offending and its associated social and economic costs.

